# An objective assessment of the variability in number of drops per bottle of glaucoma medication

**DOI:** 10.1186/s12886-017-0473-8

**Published:** 2017-05-22

**Authors:** Daniel B. Moore, Judy Beck, Richard J Kryscio

**Affiliations:** 1 Department of Ophthalmology and Visual Sciences, University of Kentucky, 740 S. Limestone, Lexington, KY 40536 USA; 2 Departments of Biostatistics and Statistics, University of Kentucky, Lexington, KY USA

**Keywords:** Glaucoma, Pharmacology, Therapeutics, Eyedrop, Compliance, Bottle

## Abstract

**Background:**

The purpose of this study is to evaluate the number of eyedrops available per bottle of a variety of commonly prescribed glaucoma medications.

**Methods:**

Six bottles of each glaucoma medication were tested: three each in the vertical and horizontal orientations. Bottles were housed in a customized force gauge apparatus designed to mimic ballpoint fingertip contact with a bottle. At a standard rate, all drops were expressed from each bottle and counted with an automated drop counter. Simultaneously, bottle volume was measured and drop size and number were also estimated. The main outcome measures were: total number of drops, volume per bottle and drops per milliliter (mL) of glaucoma medication.

**Results:**

A total of 192 bottles from 32 bottle designs and manufacturers were tested. Twenty-two of the 32 bottle designs had a significantly different mean number of drops in the vertical and horizontal positions, with 10 designs have more drops dispensed in the horizontal orientation and 12 in the vertical orientation. Six of the 32 bottle designs had a significantly different mean total bottle volume in the vertical and horizontal positions, with all designs having greater volume in the vertical position. An adjusted ratio of mean number of drops/mean bottle volume demonstrated a range from 20.9 drops/mL to 40.8 drops/mL.

**Conclusions:**

There is significant variability in drops and volume available per bottle of glaucoma medication depending on both the bottle position and manufacturer. These data point to the need for circumspection in prescribing glaucoma medications and caution in evaluating therapeutic outcomes.

## Background

Although data demonstrate improved outcomes with lowered intraocular pressure from appropriate pharmacotherapy [1, 2], many patients with glaucoma struggle to adhere to their prescribed regimens. Several studies suggest patients comply with 70% or fewer of their glaucoma medications [3–5]. Many factors have been implicated [6], but difficulty instilling drops has garnered recent attention [7]. Proper eyedrop administration requires eye-hand coordination and dexterity, linking visual acuity with a steady hand and accurate proprioception [8]. Not surprisingly, videotape evidence demonstrated that glaucoma patient used an average of 1.4–1.8 drops when trying to instill a single eyedrop [9, 10]. A recent cross-sectional patient survey revealed that 25% of patients reported problems with early eyedrop bottle exhaustion and associated compliance with therapy. One-third of patients reported the reason for early bottle exhaustion was due to bottle related problems such as “more than one drop comes out” or “size of drops is too large”. The latter complaint is supported by literature that demonstrates the volume of an eyedrop in an ophthalmic solution may vary from 25 to 70 μl [11]. Given the normal tear film volume is 7 μl and only capable of containing 30 μl without overflow, a significant portion of an eyedrop is wasted [12]. These data suggest that a significant number of glaucoma patients run out of eyedrops prior to a scheduled prescription refill and that bottle related mechanics play a role [13].

Despite the importance of bottle design in proper use of topical therapeutics, there exists no standardization of manufacture in regards to drop instillation dynamics [14], and the only dosing requirement is to accurately label and package medication volume [15]. Being manufactured and sold on the basis of volume, eyedrops are distinct from most other forms of pharmacotherapy, which are prescribed with a discrete number of doses to guide dispensing and refill rates. While the minimum volume of medication consumers should anticipate per container of medication is available, this does not necessarily translate to number of applications, and without regulation, leads to the possibility of inconsistency in the number of drops of medicine available per bottle. Several previous studies evaluating a smaller number of medications in a non-standardized fashion have found significant variability in the number of drops between both brands of medication and position of the bottle [16–18]. As such, the current study was designed to further evaluate this possibility by objectively and systematically measuring the number of eyedrops in each bottle of many common glaucoma medications.

## Methods

**Fig. 1:**
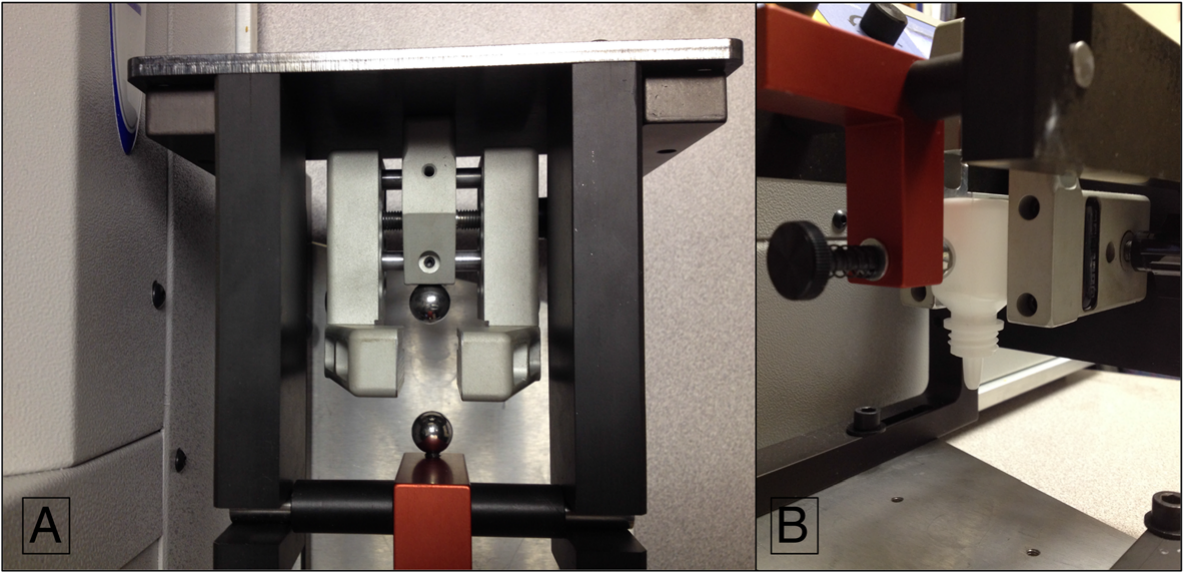
Force Gauge Apparatus. A force gauge apparatus consisting of a Mecmesin M500E Motorized Tension and Compression Stand, Mecmesin 100 N Advanced Force Gauge and custom grips and compressors were designed and calibrated by JA King & Company. **a**: The compressors were designed to mimic ballpoint fingertip contact with a bottle tip. **b**: For each medication, the bottle was housed in the apparatus and clamps were adjusted until the ballpoint compressors were located at mid bottle length. The L-shaped compression clamp was then adjusted until the force gauge sensor was centered on the crosshairs of the clamp at a 90-degree angle

The number of eyedrops dispensed from various common glaucoma medications was measured. All medications were purchased at cost from the University of Kentucky Research Pharmacy and represented available regional brand and generic medications. A force gauge apparatus consisting of a Mecmesin M500E Motorised Tension and Compression Test Stand, Mecmesin 100 N Advanced Force Gauge (Mecmesin Corporation, Sterling, VA, USA) and custom grips and compressors were designed and calibrated by JA King & Company (Whitsett, NC, USA) (Fig. 1). The compressors were designed to mimic ballpoint fingertip contact with a bottle. For each medication, the bottle was housed in the apparatus and clamps were adjusted until the ballpoint compressors were located at mid bottle length. For bottles with a rectangular instead of round shape, the thinner dimensions were chosen for compression, as this represents the method most likely to be utilized by patients when instilling drops. Starting at 0 kg-force (kgf) and 0 mm (mm) displacement, the gauge was advanced in 0.1 mm increments until a drop of liquid fell from the bottle, as observed subjectively and confirmed with an automated VCD-BTD drop counter (Vernier Software and Technology, Beaverton, OR, USA) and LabQuest 2 display (Vernier Software and Technology, Beaverton, OR, USA). At a rate of approximately one drop/s, 10 drops were expressed, then the apparatus was retracted to 0 kgf. This was repeated until all drops were exhausted from the bottle.

Simultaneously, drop size and number was also estimated using the densitometric method for volume determination [19]. In twenty drop increments, the total volume of solution expressed was measured with a 0.0001 g analytical balance (Ohaus Corporation, Parsippany, NJ, USA). This was repeated until all drops were exhausted from the bottle. A 200 uL pipette (Zhejiang Huawei Scientific Instrument Co, LTD, Zhe Jiang, China) was used to remove four 100-uL aliquots of each bottle. The mean of the samples was divided by 0.1 mL to estimate the volume of each drop and each bottle by dividing the mass of each by the calculated density. For any bottles with residual liquid in the container lid, this was removed with the pipette and volume was measured separately.

Six bottles of each medication were tested. Three bottles were tested in the vertical orientation with the bottle tip at 180 degrees and three bottles were tested in the near horizontal orientation with the bottle tip at 30 degrees. The vertical and horizontal orientations were the starting position for the bottle tip during each measurement, as compression of the bottle variably and slightly changed the tip position.

### Statistical analysis

Mean response was compared by constructing an analysis of variance for a two way layout with factors: position (horizontal versus vertical) and bottle (all combinations of medication name and formulation). A highly significant interaction between position and bottle was obtained (*p* < 0.0001). Post hoc comparison of means was done by comparing means between positions for each bottle by using two sample t-tests. To compare different bottle designs in the same orientation, Fisher’s least significant differences allowance was computed. Statistical significance was determined at the 0.01 level to minimize the Type I error rate.

## Results

A total of 192 bottles from 32 bottle designs and manufacturers of medication were tested (Table 1). Further reference to medications will include brand name, manufacturer if more than one generic and bottle volume for identification. Fifteen formulations came from the same lot, sixteen came from a combination of two different lots and one came from a combination of three different lots. When comparing the mean value of variance between formulations from the same or different lots, there was no statistical difference (*p* = 0.62). Comparing mean number of drops per bottle using observed measurements versus the densitometric method to calculate number of drops in the bottle, there was no difference in the vertical (*p* = 0.35, paired t-test), but a significant difference between measurements in the horizontal (*p* = 0.02, paired t-test) position. Observed measurements via the automated drop counter were used for subsequent analysis.

**Table 1:** Description of Glaucoma Medications Tested

Medication Name	Formulation	Manufacturer
2.5 ml		
Travatan	travaprost 0.004%	Alcon Laboratories, Inc. Fort Worth, TX 76134
Travaprost	travaprost 0.004%	Par Pharmaceutical Cos. Inc. Spring Valley, NY 10977
Xalatan	latanoprost 0.005%	Pharmacia&Upjohn Co, Division of Pfizer, Inc. NY, NY 10017
Latanoprost	latanoprost 0.005%	Akorn, Inc. Lake Forest, IL 60045
Lumigan	bimatoprost 0.001%	Allergan, Inc. Irvine, CA 92612
5 ml		
Lumigan	bimatoprost 0.001%	Allergan, Inc. Irvine, CA 92612
Travaprost	travaprost 0.004%	Par Pharmaceutical Cos. Inc. Spring Valley, NY 10977
Travatan	travaprost 0.004%	Alcon Laboratories, Inc. Fort Worth, TX 76134
Alphagan P 0.1%	brimonidine tartrate 0.1%	Allergan, Inc. Irvine, CA 92612
Combigan	brimonidine tartrate 0.2%, timolol maleate 0.5%	Allergan, Inc. Irvine, CA 92612
Timolol Pacific	timolol maleate 0.5%	Pacific Pharma, Irvine CA, 92,612
Timolol Hi-Tech	timolol maleate 0.5%	Hi-Tech Pharmacal Co, Inc. Amityville, NY 11701
Timolol Sandoz	timolol maleate 0.5%	Alcon Laboratories, Inc. Fort Worth, TX 76134 for Sandoz Inc. Pricenton, NJ 08540
Betimol	timolol maleate 0.5%	Akorn, Inc. Lake Forest, IL 60045 for Oak Pharmaceuticals, Inc.
Timoptic XE	timolol maleate 0.5%	Merck Sharp & Dohme-Chibret 63,963 Clermont-Ferrand Cedex 9, France; Distributed by Valeant Ophthalmics, a division of Valeant Pharmaceuticals North America LLC, Bridgewater, NJ 08807
Timoptic	timolol maleate 0.5%	Merck Sharp & Dohme-Chibret 63,963 Clermont-Ferrand Cedex 9, France; Distributed by Valeant Ophthalmics, a division of Valeant Pharmaceuticals North America LLC, Bridgewater, NJ 08807
Istalol	timolol maleate 0.5%	Bausch&Lomb Incorporated Tampa, FL 33637 under License from Senju Pharmaceutical Co, Ltd. Osaka, Japan 541–0046
7.5 ml		
Lumigan	bimatoprost 0.001%	Allergan, Inc. Irvine, CA 92612
8 ml		
Simbrinza	brinzolamide 1%, brimonidine tartrate 0.2%	Alcon Laboratories, INC 6201 South Freeway, Fort Worth, TX 76134
10 ml		
Cosopt	dorzolamide HCL 22.3 mg/ml, timolol maleate 6.8 mg/ml	Akorn, Inc. Lake Forest, IL 60045
Dorz/Tim Hi-Tech	dorzolamide HCL 22.3 mg/ml, timolol maleate 6.8 mg/ml	Hi-Tech Pharmacal Co, Inc. Amityville, NY 11701
Dorz/Tim Bausch	dorzolamide HCL 22.3 mg/ml, timolol maleate 6.8 mg/ml	Bausch & Lomb Incorporated Tampl, FL 33637
Dorz/Tim Sandoz	dorzolamide HCL 22.3 mg/ml, timolol maleate 6.8 mg/ml	Alcon Laboratories Inc. Fort Worth Tx 76,134 for Sandoz Inc. Princeton, NJ 08540
Trusopt	dorzolamide HCL 2%	Merck Sharp & Dohme Corp.,a subsidiary of Merck & Co, Inc. Whitehouse Station, NJ 08889
Dorz Hi Tech	dorzolamide HCL 2%	Hi-Tech Pharmacal Co, Inc. Amityville, NY 11701
Dorz Bausch	dorzolamide HCL 2%	Bausch & Lomb Incorporated Tampa, FL 33637
Dorz Teva	dorzolamide HCL 2%	Teva Pharmaceutical Ind, Ltd. Jerusalem, 91,010, Israel for Teva Pharmaceuticals USA Sellersville, PA 18960
Dorz Sandoz	dorzolamide HCL 2%	Alcon Laboratires, Inc. Fort Worth, TX 76134 for Sandoz Inc. Princeton, NJ 08540
Azopt	brinzolamide 1%	Alcon Laboratories, Inc. Fort Worth, TX 76134
Combigan	brimonidine tartrate 0.2%, timolol maleate 0.5%	Allergan, Inc. Irvine, CA 92612
Alphagan P 0.15%	brimonidine tartrate 0.15%	Allergan, Inc. Irvine, CA 92612
Alphagan P 0.1%	brimonidine tartrate 0.1%	Allergan, Inc. Irvine, CA 92612

The medication name and volume used in the study, the formulation and manufacturer are listed

The mean number and standard deviation of drops per bottle in the horizontal, vertical and summative positions of all formulations are provided in Table 2. For 2.5 mL bottles, the mean number of drops ranged from 75.3–101.7 and 72–102.3 in the vertical and horizontal orientations, respectively. For 5 mL bottles, the range was 111–209.3 and 115–189 drops in the vertical and horizontal orientations, respectively. For 10 mL bottles, the range was 193.7–313.3 and 234–323.7 drops in the vertical and horizontal orientations, respectively. Twenty-two of the 32 bottle designs had a significantly different mean number of drops in the vertical and horizontal positions, with 10 designs have more drops dispensed in the horizontal orientation and 12 in the vertical.

**Table 2:** Mean Number of Drops, Volume and Drop/Volume Ratio per Glaucoma Medication Tested

Drop	Mean # Drops			Mean Bottle Volume			Mean Drop Volume		Mean Drops/mL		
	vert	horz	*p* value	vert	horz	*p* value	vert	horz	vert	horz	*p* value
2.5 ml											
Travatan	76.3 ± 4.2	102.3 ± 1.5	**<0.01**	2.57 ± 0.07	2.53 ± 0.04		0.032	0.025	29.66 ± 1.35	40.4 ± 1.07	**0.0004**
Travaprost	75.3 ± 5.1	72 ± 1		2.46 ± 0.04	2.36 ± 0.07		0.032	0.032	30.6 ± 2.51	30.47 ± 1.14	0.9445
Xalatan	101.3 ± 0.6	83.3 ± 3.1		2.69 ± 0.05	2.70 ± 0.05		0.027	0.032	37.68 ± 0.73	30.90 ± 0.60	**0.0002**
Latanoprost	86.7 ± 9.3	76.7 ± 5.0		2.37 ± 0.26	2.42 ± 0.13		0.027	0.032	36.59 ± 0.98	31.63 ± 0.76	**0.0023**
Lumigan	101.7 ± 2.3	81.7 ± 4.0	**<0.01**	2.50 ± 0.05	2.38 ± 0.08		0.024	0.029	40.61 ± 0.18	34.25 ± 0.63	**<0.0001**
overall mean	88.3	83.2		2.52	2.48		0.029	0.03			
overall median	91	81		2.53	2.46		0.027	0.031			
overall ST DEV	12.7	11.08		0.15	0.15		0.004	0.003			
5 ml											
Lumigan	195.6 ± 4.2	155 ± 1	**<0.01**	4.97 ± 0.06	4.73 ± 0.06		0.025	0.03	39.4 ± 0.69	32.75 ± 0.22	**<0.001**
Travaprost	150.3 ± 6.5	139.7 ± 13.7		4.91 ± 0.02	4.78 ± 0.24		0.032	0.035	30.62 ± 1.42	29.15 ± 1.50	0.2859
Travatan	146.3 ± 0.6	189 ± 1	**<0.01**	4.8 ± 0.04	4.78 ± 0.07		0.033	0.026	30.49 ± 0.82	29.66 ± 1.35	**0.0004**
Alphagan P	111 ± 1	117 ± 2.6		4.89 ± 0.05	4.72 ± 0.18		0.044	0.04	22.7 ± 0.16	24.82 ± 0.78	**0.0102**
Combigan	148 ± 2	161.3 ± 3.8		5.02 ± 0.16	4.67 ± 0.07	**0.01**	0.033	0.029	25.26 ± 1.53	34.57 ± 0.50	**0.0003**
Timolol Pacific	123 ± 1	147.7 ± 9.6	**<0.01**	4.88 ± 0.04	4.69 ± 0.10		0.04	0.031	25.21 ± 0.09	25.21 ± 0.09	0.0206
Timolol Hi-Tech	126.7 ± 6.7	149.3 ± 4.0	**<0.01**	5.09 ± 0.15	5.04 ± 0.13		0.041	0.034	24.89 ± 0.56	29.61 ± 0.633	**0.0006**
Timolol Sandoz	161.3 ± 3.5	156.3 ± 2.5		4.89 ± 0.04	4.84 ± 0.08		0.03	0.031	33.02 ± 0.92	32.28 ± 0.21	0.2459
Betimol	165.3 ± 0.6	160.7 ± 1.2		5.17 ± 0.035	5.08 ± 0.06		0.031	0.031	32.00 ± 0.14	31.63 ± 0.56	0.3282
Timoptic XE	130.7 ± 7.5	115 ± 9.8	**0.004**	5.53 ± 0.24	4.9 ± 0.24		0.042	0.043	23.71 ± 2.41	23.45 ± 1.16	0.8734
Timoptic	209.3 ± 5.7	155.3 ± 7.1	**<0.01**	5.93 ± 0.03	5.66 ± 0.03	**0.0003**	0.089	0.036	35.28 ± 0.82	27.6 ± 1.21	**0.0008**
Istalol	154.6 ± 2.1	165.6 ± 2.5		4.72 ± 0.03	4.74 ± 0.09		0.03	0.029	32.77 ± 0.22	34.95 ± 0.32	**0.0006**
overall mean	151.9	151		5.06	4.89		0.039	0.033			
overall median	149	155.2		4.93	4.78		0.033	0.031			
overall ST DEV	28.2	20.26		0.34	0.28		0.03	0.005			
7.5 ml											
Lumigan	294 ± 2.6	236 ± 2	**<0.01**	7.21 ± 0.06	7.18 ± 0.03		0.024	0.03	40.76 ± 0.56	32.85 ± 0.18	**<0.001**
8 ml											
Simbrinza	180.7 ± 3.1	219.7 ± 6.7	**<0.01**	7.51 ± 0.03	6.74 ± 0.12	**0.0004**	0.167	0.031	24.07 ± 0.47	32.58 ± 1.22	**0.0003**
10 ml											
Cosopt	301.7 ± 39.6	242 ± 12.5	**<0.01**	10.2 ± 0.17	9.58 ± 0.18	**0.01**	0.033	0.039	29.56 ± 3.66	25.26 ± 1.53	0.1341
Dorz/Tim Hi-Tech	303 ± 1	251.7 ± 10.0	**<0.01**	9.23 ± 0.34	9.19 ± 0.26		0.031	0.038	32.85 ± 1.22	27.39 ± 0.39	**0.0018**
Dorz/Tim Bausch	313.3 ± 1.5	293.7 ± 1.5	**<0.01**	9.22 ± 0.23	8.91 ± 0.21		0.03	0.031	34.01 ± 0.92	32.96 ± 0.65	0.1804
Dorz/Tim Sandoz	315 ± 4.6	271.3 ± 5.8	**<0.01**	8.93 ± 0.15	8.39 ± 0.29		0.115	0.031	35.27 ± 1.09	32.37 ± 1.03	0.0288
Trusopt	313.3 ± 20.0	236 ± 5.6	**<0.01**	10.18 ± 0.20	9.60 ± 0.33		0.221	0.051	30.77 ± 1.86	24.59 ± 0.94	**0.0068**
Dorz Hi Tech	292.7 ± 10.3	261.7 ± 12.9	**<0.01**	9.54 ± 0.19	9.13 ± 0.29		0.033	0.035	20.92 ± 17.91	28.70 ± 2.21	0.5313
Dorz Bausch	289.7 ± 4.2	289 ± 4.6		9.18 ± 0.26	8.78 ± 0.15		0.032	0.03	31.55 ± 0.61	32.91 ± 0.06	0.0594
Dorz Teva	282 ± 11.5	260 ± 1	**<0.01**	9.31 ± 0.58	8.69 ± 0.12		0.033	0.034	30.33 ± 0.79	29.91 ± 0.31	0.4423
Dorz Sandoz	193.7 ± 3.79	253.7 ± 15.3	**<0.01**	9.19 ± 0.12	8.38 ± 0.08	**0.0006**	0.332	0.034	21.06 ± 0.16	30.26 ± 1.94	0.014
Azopt	254.3 ± 2.1	322 ± 8.9	**<0.01**	9.42 ± 0.08	8.59 ± 0.04	**<0.0001**	0.037	0.027	27.01 ± 0.38	37.5 ± 1.08	**<0.001**
Combigan	270.3 ± 28.4	323.7 ± 17.8	**<0.01**	9.86 ± 0.21	9.79 ± 0.07		0.034	0.03	27.4 ± 2.50	33.07 ± 1.64	0.0303
Alphagan 0.15%	212.7 ± 3.1	234 ± 3.6	**<0.01**	9.78 ± 0	9.53 ± 0.09		0.046	0.041	21.75 ± 0.31	24.56 ± 0.16	**0.0002**
Alphagan 0.1%	216 ± 4	283.3 ± 5.5	**<0.01**	9.93 ± 0.19	9.61 ± 0.07		0.046	0.037	21.75 ± 0.11	24.80 ± 0.41	**0.0002**
overall mean	273.7	267.5		9.54	9.09		0.079	0.034			
overall median	290	261		9.51	9.02		0.034	0.033			
overall ST DEV	42.69	31.07		0.45	0.51		0.119	0.005			

The mean number of drops, bottle volume, drop volume and ratio of mean number of drops per bottle volume of three bottles tested for each medication in the vertical and horizontal positions. Medications are grouped by volume and an overall mean, median and standard deviation for all medications of similar volumes are provided. A *p*-value for medications with a significant difference in mean drop number between the horizontal and vertical position is listed, with significance determined at *p* = 0.01. A ratio of mean number of drops/mean bottle volume is listed, and any two means that differ by a value of 5.27 or greater are significantly different (*p* < 0.01). Volume measurements are in milliliters (mL). A bold value indicates a *p* value of statistical significance

The mean bottle and drop volume are also listed in Table 2. Bottle volumes were measured with and without accounting for residual volume in the cap of the container, with less than a 2.2% difference of the overall mean in 90% of the bottles. Presented bottle volume measurements include the cap volume. For 2.5 mL bottles, the range was 2.37–2.69 mL and 2.38–2.70 mL in the vertical and horizontal orientations, respectively. For 5 mL bottles, the range was 4.72–5.93 mL and 4.67–5.66 mL in the vertical and horizontal orientations, respectively. For 10 mL bottles, the range was 8.93–10.2 mL and 8.38–9.79 mL in the vertical and horizontal orientations, respectively. Six of the 32 bottle designs had a significantly different mean total bottle volume in the vertical and horizontal positions, with all designs having greater volume in the vertical position. There were no significant differences between the horizontal and vertical measured drop volume for any designs.

Given the multiple different comparisons possible between bottle type, size and volume, an adjusted ratio of mean number of drops/mean bottle volume was created (Table 2), with a range from 20.9 drops/mL to 40.8 drops/mL among the bottle designs and positions. Nineteen formulations had a significantly different ratio between the horizontal and vertical positions (*p* < 0.01). For comparing means between two bottle designs in the same orientation, any two means that differ by a value of 5.27 or greater are significantly different (*p* < 0.01).

## Discussion

The results of this study suggest there is significant variability in the number of drops and volume per bottle of glaucoma medications, both in terms of which bottle design and manufacturer is utilized and the position the bottle is held when squeezed. For example, if a representative patient from this region is prescribed a 5 mL bottle of timolol 0.5%, there are 5 different generic and brand bottle designs that may be dispensed. The patient could anticipate a range of 123–209 and 147–166 mean drops per bottle in the horizontal and vertical positions, respectively. If instilled twice daily, this would suggest a difference between 25.5 to 43 days of available medication depending on the manufacturer and bottle position.

This study is the first to our knowledge to present an objective, automated and reproducible method to measure the number of drops available per bottle of medication. Further, we measured countable drops instead of calculating the number of drops based on volume, which was shown to be inaccurate. Several previous studies have evaluated small samples of bottle formulations with similar, variable results. A 1994 study of patients blinded to either use of a 5 mL bottle of timolol maleate versus levobunolol found a 21% greater length of use of timolol (37 versus 29 days) [16]. Another study evaluated 45 versus 90 degree administration of artificial tear bottles and found a significantly greater number of drops per bottle using densitometric analysis and smaller drop volume for 4 out of 5 formulations at 45 compared to 90 degrees. The authors suggest administration at 45 degrees would result in up to $1.93 savings per bottle compared to 90 degrees [17]. A study evaluating 2.5 ml bottles of prostaglandin analogues held vertically, at 45 degrees and horizontally found that vertical instillation resulted in more drops per bottle for bimatoprost and latanoprost, while 45 degrees was most efficient for travaprost. Assuming 1 year of bilateral therapy at 2006 costs, the authors determined use of the most efficient instillation method would result in yearly savings of $109–192 [18]. Lastly, a recent study found significant variability in the number of eyedrops per bottle of four regionally available formulations of latanoprost when measured by manually counting the number of drops expressed by hand, ranging from 77.6 to 88.7 drops per bottle. The authors estimated a similarly significant difference in estimated annual cost, ranging from $184 to $1198 per formulation [20].

Currently, there exist no federal guidelines to regulate bottle design or amount of drops available per volume of medication [14, 15]. It has been suggested that pharmacists often use a rough guideline of 0.05 mL per eyedrop or 20 drops per mL (written personal communication, Division of Drug Information, FDA, March 27, 2015). With that in mind, there are significantly more eyedrops per bottle in this analysis than recommended, ranging from 10.4% to 45.8% more mean drops per bottle tested. This may indicate that manufacturers “overfill” the bottles to allow for a margin of error during dispensing. If true, this serves an important purpose, since it is known that many patients require more than one eyedrop per application [9, 10]. However, this data suggests another problem has been created by this practice: significant variability in the number of doses per bottle.

Aside from bottle volume, an additional factor influence the number of drops available per bottle is drop size. We found significant variability in the estimated drop size of studied formulations, ranging from 0.024 to 0.221 mL. The size of drop dispensed from a bottle depends on three basic elements: the design of the dropper bottle and tip, properties of the contained solution and the position of the bottle. The surface area around the bottle tip and surface tension of the solution are both manufacturer controlled factors that influence drop size. Patient manipulations such as the angle and rate drops are produced are less predictable [11]. Further, the results of the current study and others suggest the most economical bottle position varies from one design to the next [17–19]. A final potential determinant of drop size is the force required to squeeze the bottle, which unfortunately is significantly variable in both the experimental and clinical setting [21, 22]. With all of these influences, it may be difficult to design an ideal bottle for instillation. One expert suggestion has been to utilize a dropper tip with a smaller outer orifice diameter that provides consistent surface area for a smaller-volume drop to fall [11].

This study has several limitations. The experimental design was novel, and although measurements were automated, they have not been independently verified. Because the study was conducted in an objective and reproducible manner, it may not accurately reflect many of the patient related factors in dosing; it is likely that our patients experience even greater variability in the number of drops available per bottle. Only two bottle positions were tested and most ophthalmic containers are not intended to be delivered in a strictly horizontal position. While 192 bottles from 32 bottle designs were tested, they still represent a small sampling of all available brand and generic ophthalmic medications.

## Conclusion

In summary, this study demonstrates the significant variability in drops and volume available per bottle of glaucoma medication depending on both the bottle position and manufacturer. This unregulated practice leaves prescribing physicians and pharmacists unable to accurately predict the quantity of medication to dispense. This may lead to patients running out of medication early or being left with excess and associated costs. The variability from one refill to the next could be a contributor to limited compliance. The experimental design in this study indicates an objective, reproducible method to determine drop number uniformly across different bottles and designs. It should compel further evaluation and consideration of standardization in the industry.

## Abbreviations

mL: milliliter
mm: millimeter
μl: microliter
